# Permeation of Ternary Mixture Containing H_2_S, CO_2_ and CH_4_ in Aquivion^®^ Perfluorosulfonic Acid (PFSA) Ionomer Membranes

**DOI:** 10.3390/membranes12111034

**Published:** 2022-10-24

**Authors:** Virginia Signorini, Marco Giacinti Baschetti, Diego Pizzi, Luca Merlo

**Affiliations:** 1 Department of Civil, Chemical, Environmental and Material Engineering (DICAM), Alma Mater Studiorum, University of Bologna, Via Terracini 28, 40131 Bologna, Italy; 2 Renco S.p.A., V.le Venezia 53, 61122 Pesaro, Italy; 3 Solvay Specialty Polymers Italy S.p.A., V.la Lombardia 20, 20021 Bollate, Italy

**Keywords:** Aquivion^®^ PFSA, gas permeation, H_2_S, CO_2_, natural gas sweetening, ternary mixture

## Abstract

Aquivion^®^ E87-12S Perfluorosulfonated acid ionomer material (PFSA) has been studied as a membrane technology for natural gas sweetening from CO_2_, H_2_S due to its interesting chemical and mechanical stability and good separation performance for polar compounds in humid environments. In the present work, permeation of the H_2_S/CO_2_/CH_4_ ternary mixture in this short-side PFSA chain was investigated at pressures up to 10 bar, temperatures up to 50 °C, and in a range of relative humidity (RH) from 20% to 90%. The results obtained confirm the strong dependence of Aquivion^®^ on water activity and temperature, and its ability to separate gases based on their water solubility without substantial differences between pure and mixed gas experiments. Indeed, even when tested in ternary mixture, the permeation behavior remains similar to that observed for pure components and binary mixtures. In particular, the permeability of H_2_S is higher than that of CO_2_ and methane CH_4_, reaching values of 500 Barrer at 50 °C and 80% RH, against 450 and 23 Barrer for the other two gases respectively. Additionally, when tested at higher pressures of up to 10 bar under humid conditions, the membrane properties remained largely unchanged, thus confirming the overall stability and durability of Aquivion^®^ E87-12S in acid environments.

## 1. Introduction

Energy demand has increased enormously over the last century, making natural gas (NG) one of the most environmentally friendly fossil fuels. The raw NG stream mainly contains methane (>70%) together with other undesirable impurities, such as H_2_S, CO_2_, water and higher hydrocarbons that must be removed prior to commercialization [1]. In particular, hydrogen sulfide (H_2_S) and carbon dioxide (CO_2_) are the best known acidic compounds present in natural gas, and their presence is harmful for both environmental and safety reasons. H_2_S is a highly toxic compound that is corrosive for equipment and pipelines, especially in the presence of water vapor, and CO_2_ decreases the heating values of the methane stream, and it is the green-house gas most responsible for the negative effects of global warming [1,2]. Their presence must therefore be reduced in natural gas streams in order to meet pipeline specifications and to make it more attractive and suitable for fulfilling, in a sustainable way, energy demand, which is expected to grow by over 50% by the year 2035 [3].

For this purpose, the selection of a method for the separation of acid gas from natural gas should meet the requirements of gas stream performance, physical and chemical conditions, and economic demands [4]. Moreover, in recent years, the sustainability of the approach has also become one of the main factors influencing this choice [5].

Membrane technology is a relatively new method of gas separation when compared to classical technologies such as absorption or distillation, as its industrial applications started only in the second half of the last century [6]. Since then, polymeric membrane technology has become one of the most studied methods of gas separation because of its good performance, high efficiency in gas transport, mechanical and operational simplicity, and relatively low cost of production and manufacturing [6,7,8].

However, there is a limited range of polymers that can be used for natural gas sweetening due to the poor separation efficiency of most materials and their relatively low resistance to humidity, along with problems like plasticization and/or competitive sorption, which decrease and limit the performances and the durability of membranes under real operating conditions [7,9,10,11]. The research has grown substantially, especially regarding the use of glassy polymers to perform membrane separation processes for the removal of H_2_S from natural gas with the aim of using this type of material in high-pressure applications [12,13]. Moreover, glassy polymer membranes in dense film forms have also shown a greater degree of control over the plasticization effect, swelling and competitive sorption, resulting in a higher permeability of CO_2_ and H_2_S, together with an increase in acid/methane selectivity [14]. This innovative development has resulted in Robeson’s upper bound limit being updated, both for CO_2_/CH_4_ and H_2_S/CH_4_, thus proposing a new benchmark for the development of membrane technology [15]. In this regard, among the various possibilities, perfluorinated materials are emerging as an optimal class for gas separation membranes, especially in the presence of water vapor [16,17,18]. Thanks to their strong C-F bond energy, perfluorinated polymers exhibit excellent chemical and mechanical stability, together with extremely good resistance to plasticization induced by CO_2_ and H_2_S compounds [16]. In particular, sulfonic ionomers such as Nafion^®^ and Aquivion^®^ are a peculiar class of perfluorinated polymers, since they demonstrate a very interesting affinity with respect to polar compounds and good separation performance under humid conditions [17,19,20,21]. Perfluorosulfonated acid ionomer polymers (PFSA) are made of hydrophobic fluorinated backbone and hydrophilic sulfonic end groups that are able to absorb water [17,22]. This heterogeneous structure allows, upon polymer hydration, the formation of water-channel domains inside the matrix, which become the preferential pathway for the diffusion of the gases [20,23,24]. Many recent works [19,21,25,26,27,28] have studied the separation performances of the semi-commercial polymeric material Aquivion^®^, which has been characterized in dry and humid conditions with various gases, such as CO_2_, CH_4_, H_2_S, C_3_H_8_ and C_4_H_10_, at different temperatures. From the results present in the literature, it can be observed that both water and temperature positively affect the separation properties of this type of material: in fact, the permeabilities of all gases increase by more than two orders of magnitude when passing from the dry test to testing in a humid environment, while still maintaining the ideal selectivity behavior [19,28]. PSFA would therefore be of interest for the treatment of natural gas streams, which are often close to saturation when extracted from the wells [4,29]. 

Despite the amount of research performed on gas permeability in PSFA, to date, very little information has been reported on the behavior of materials in the presence of ternary mixtures [26,30,31], and none of them actually refers to mixtures containing H_2_S in Aquivion^®^. Specifically, some papers have already been published about binary permeation tests, but the simultaneous presence of both CO_2_ and H_2_S in the feed stream has not been investigated. This is an important feature to look at, especially with regard to the presence of competitive sorption between the two acid gases, as has previously been observed in other similar materials [32]. In order to partially fill this gap and to clarify the possible interaction of acidic gases in the material, in this work, the Aquivion^®^ E87-12S membrane was characterized using a ternary mixture made of 10% CO_2_, 10% H_2_S and 80% CH_4_, at three different temperatures—25 °C, 35 °C and 50 °C—and relative humidity ranging from 20% to 95%. In addition, in order to investigate its real behavior when subjected to a high partial pressure of acid gas, the material was tested at 35 °C with a total feed pressure of up to 10 bar and water relative humidity (RH) equal to 50% and 90%. These data made it possible to better understand the membrane’s behavior in situations similar to industrial conditions, since, in general, natural gas is treated at high pressure and at high RH [13,33,34].

## 2. Materials and Methods

**Figure 1:**
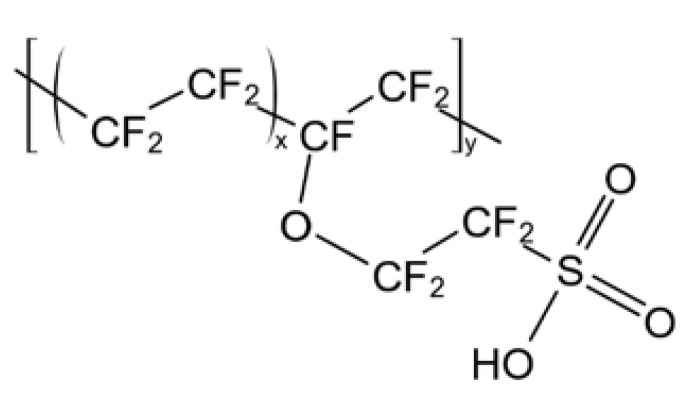
Chemical structure of Aquivion^®^.

The material used for the studies on ternary mixture permeation is a commercial perfluorosulfonic acid (PFSA) ionomer membrane, produced by Solvay Specialty Polymers Italy S.p.A. (Bollate (MI) Italy) using a melt-extrusion process. It is commercialized with the trade name Aquivion^®^ E87-12S, and it exhibits an average thickness of 120 µm and an equivalent weight of 870 gpol/molSO_3_H. The Aquivion^®^ E87-12S polymer has a chemical structure (Figure 1) similar to that of Nafion^®^, but with a shorter side chain and higher crystallinity, which give it higher mechanical properties and thermal resistance compared to Nafion^®^ [18,21,35]. Tests were performed using pure CO_2_, characterized by a purity of 99.99%, and a calibrated mixture of methane and hydrogen sulfide (90% and 10%vol, respectively) provided by Fluido Tecnica s.a.s (Campi Bisenzio (FI) Italy).

### Mixed Permeation Experiments

The separation properties for gas sweetening of Aquivion E87-12S were characterized by investigating the permeability of ternary mixtures containing CO_2_, H_2_S and CH_4_ measured under different operating conditions. Specifically, the tests were conducted at three different temperatures—25 °C, 35 °C and 50 °C—at relative humidities ranging from 0% to 90% and with a feed pressure varying from 2 to 10 bar.

The experiments were carried out in a purposely developed manometric system with closed volume and variable pressure, the scheme and description of which were presented in a previous work [27]. Its peculiar feature is the possibility of conducting humid and mixed gas permeability tests in a closed system, thus avoiding the waste of gases and safety problems due to the continuous feed of high-pressure toxic gases such as H_2_S. In particular, the upstream side of the membrane compartment is continuously mixed to avoid concentration polarization phenomena and a constant and equal humidity is maintained on the two sides of the membrane during the test, so that only gases pass across the membrane, and no substantial water transport occurs during the experiments, thus simplifying data processing. 

During the tests, the three investigated gases were loaded separately to the upstream tank: the CO_2_ was fed first to the desired partial pressure, then the final total pressure was reached by filling the reservoir with the pre-mixed H_2_S/CH_4_ mixture. Once the upstream compartment was filled, the permeation process was monitored by measuring the pressure increment and the mixture compositions in the calibrated downstream volume, which only depends on the flow of permeating gases across the humidified membrane.

The permeability of the three components at steady state can be calculated by comparing the experimental data with the pressure calculated by solving the mass balances in the downstream volume coupled with the ideal gas equation of state:

(1) $$\frac{dn_{i}}{dt}=-\frac{P_{i}\;\left(p_{i}^{up}-p_{i}^{down}\right)A}{l}\;\;\;i=1,\;2,\;3$$

(2) $$\frac{dp_{i}^{down}}{dt}=\frac{RT}{V_{d}}\overset{Nc}{\underset{i}{\sum }}\frac{dn_{i}}{dt}$$

where $\frac{dn_{i}}{dt}$ is the mole variation of the *i^th^* component over time, *V_d_* is the downstream volume, *A* is the membrane surface area, *l* is the membrane thickness, *T* is the fixed temperature of the system, *R* is the universal gas constant and (*p_i_^up^* *− p_i_^down^*) is the partial pressure difference of the *i^th^* component through the membrane [26].

**Figure 2:**
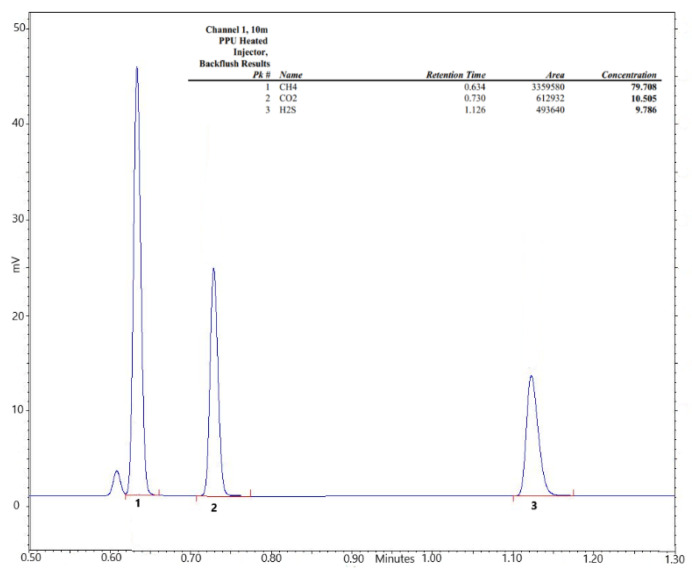
CH_4_, CO_2_ and H_2_S chromatographic peak compositions.

The proposed multiparameter fitting has three unknown variables, derived from the permeability of each component in the mixture, which were evaluated by considering the overall pressure increase in the downstream compartment together with the data related to the composition of the permeate, which were obtained using a microGC connected to the system. The GC used was a 490 Micro GC from Agilent Technology [36], equipped with a 10 m PPU adsorption column using helium as a carrier gas, and was characterized on by the following retention times for the three gases: 0.6 min for CH_4_, 0.7 min for CO_2_, and 1.1 min for H_2_S, as shown in Figure 2.

Interestingly, since Aquivion^®^ membranes are much more selective for H_2_S and CO_2_ than CH_4_, the latter gas reached equilibrium on the two sides of the membrane well after the others, which also enabled the direct calculation of methane permeability independently of the other two, thus reducing the uncertainty in the fitting procedure. By allowing the test to continue for sufficient time, methane will indeed be the only gas still permeating across the membrane, given that both CO_2_ and H_2_S are already at equilibrium. Its permeability, therefore, can be calculated using Equation (3), which is usually used for single gas permeation experiments.

(3) $$Pi={\frac{dp_{i}}{dt}|}_{s.s.\frac{V_{d}}{RTA}\frac{l}{\left(p_{i}^{up}-p_{i}^{down}\right)}}$$

where ${\frac{dp_{i}}{dt}|}_{s.s.}$ is the steady-state pressure change over time [26]. 

**Figure 3:**
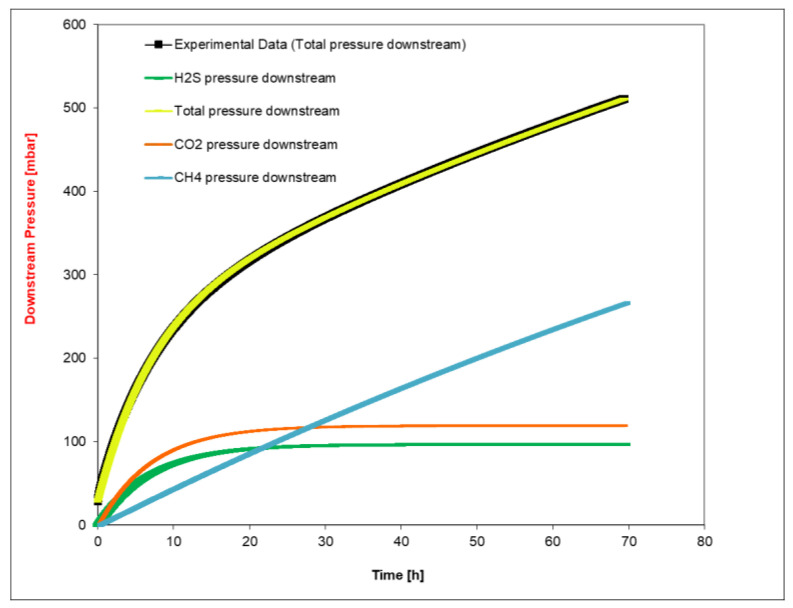
Data fitting of pressure variation over time for a H_2_S/CO_2_/CH_4_ mix gas permeation experiment.

An example of the experimental curve with the fitting results is reported in Figure 3. As can be observed, very good agreement was generally obtained; however, considering the different sources of uncertainty, an error on the order of 10% should be considered for permeability values. This estimation, which should be considered conservative, is mainly related to uncertainty with respect to humidity calibration and upstream composition, followed by temperature and pressure fluctuations and error in the measurements of film thickness. It should be noticed in particular that pressure fluctuations in the upstream side of the membranes, reported in Table 1 and mainly resulting from the loading procedures, had a minimal influence on the calculated permeability values. The properties of PFSA were indeed slightly affected by pressure below 8 bar [26].

From the permeability data obtained, it is possible to estimate the ideal selectivity as the ratio of the permeabilities of pure compounds at the same RH, as reported in Equation (4), in order to understand the real separation potential of the membrane material.

(4) $$\propto_{i/j}={\left(\frac{P_{i}}{P_{j}}\right)}_{RH}$$

## 3. Results

CO_2_/H_2_S/CH_4_ ternary mixture tests were carried out at both low and high pressure—3 bar and 10 bar, respectively—under three different temperature conditions—25 °C, 35 °C and 50 °C—and with relative humidity ranging from RH20% to RH95%. The feed stream composition was calibrated and checked using the Micro-gas Chromatograph, and it was maintained at a constant composition with molar fractions of 0.1, 0.09 and 0.8 for CO_2_, H_2_S and CH_4_, respectively.

**Figure 4:**
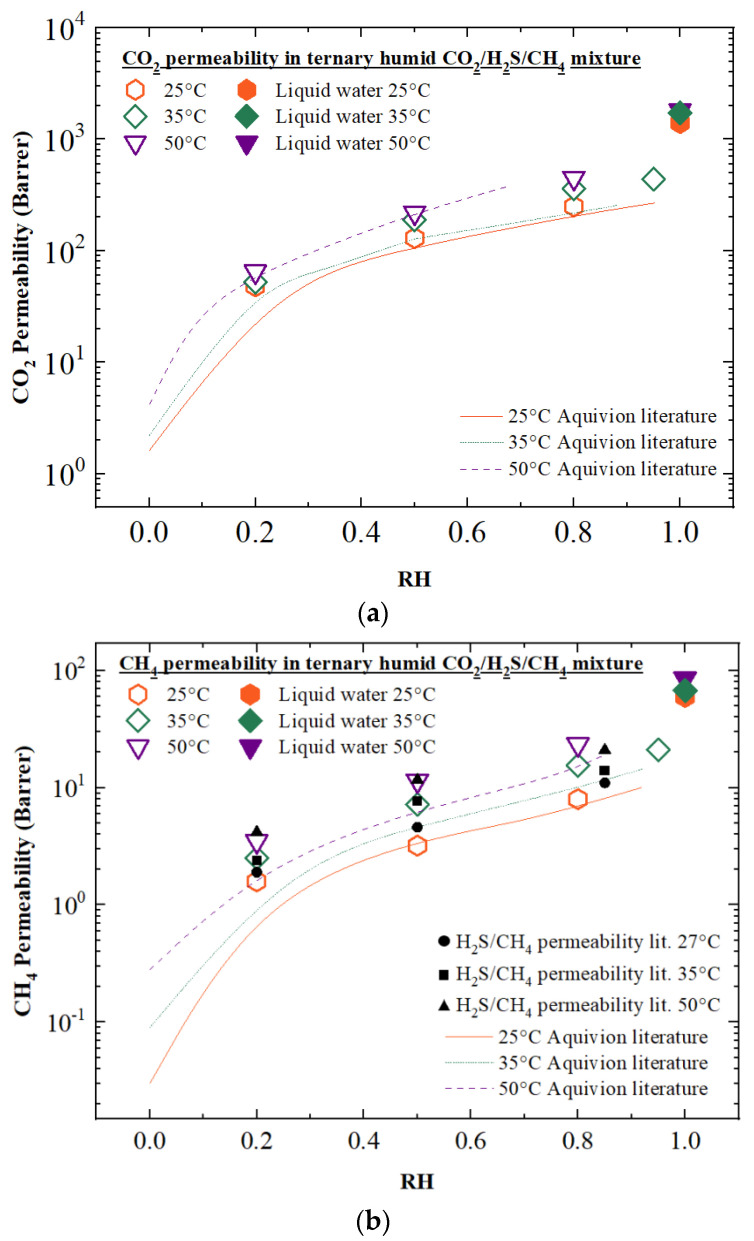

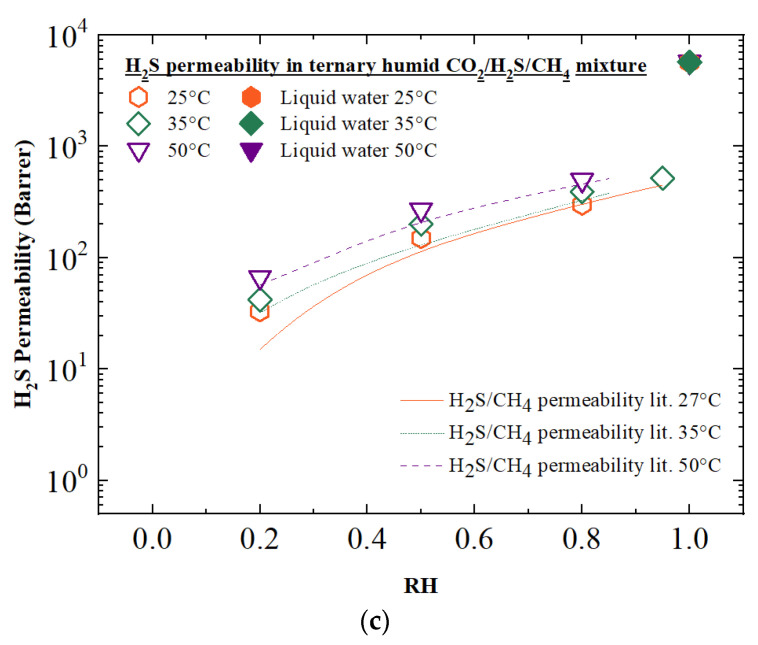
Permeability of humid (**a**) CO_2_, (**b**) CH_4_ and (**c**) H_2_S in Aquivion^®^ E87-12 S membranes compared with literature data at temperatures of 25 °C, 35 °C and 50 °C. Literature data were taken from [25] for CO_2_ and CH_4_, and from [27] for H_2_S.

The experimental results obtained are reported in Table 1 and Figure 4, where they are compared with the permeability data of pure gases [25,28] and the binary mixture test, previously presented in Ref. [27].

As can be observed from the graphs, the permeability of all the three gases tested in the ternary mixture increased with increasing temperature and relative humidity. In particular, as already observed in previous work [19,21,28], the presence of water in this kind of material leads to an increase in permeability values by up to one order of magnitude with respect to dry permeability. This is caused by the morphological change in the structure of the Aquivion^®^ upon hydration. As a result, the hydrophilic -SO_3_H groups absorb the water present in the environment, creating interconnected water-channel domains inside the matrix [21,23], making it possible for the most soluble and polar compounds to permeate more quickly across the membrane. 

The highest variation can be observed at humidity values between 0 and 20% (not investigated in this work), where the permeability of all three gases increases sharply, since water vapor saturates the membrane, and interconnected water channels start to form in the polymeric chain. With a further increase in humidity (from 20% to 95%), the permeability tends to demonstrate a more gentle rising behavior as a result of the water domains getting bigger and less tortuous [20,23].

Comparing the results with those obtained for pure components, it can be observed that the CO_2_ permeability data in the ternary mixture (Figure 4a) are slightly higher than those presented in the literature data: when the RH of the tests increases from 20% to 80%, the mix gas CO_2_ permeability at 35 °C ranges from 52 Barrer to 350 Barrer; when the test is performed with pure gas, it ranges from 45 Barrer to 255 Barrer under the same operating conditions. This effect can also be observed in methane, the permeability of which in the ternary mixture is slightly higher than the one measured for the pure component, and in line with the one observed for binary mixtures [27]. Additionally, in binary mixtures, a slight increase can be observed with respect to the pure component data, albeit more limited to the low humidity range. 

In the case of H_2_S, the results obtained for the ternary mixture permeation test were compared with previously published data for H_2_S/CH_4_ binary mixtures [27]. Indeed, to the best of our knowledge, no literature data for pure hydrogen sulfide permeability in Aquivion^®^ are currently available. Similar to what was observed for other gases, the H_2_S permeability results are in line with those previously reported for binary mixtures, with substantial deviation only being observed at low temperature, 25 °C, and low humidity (RH20%), where the permeability increases from 17 to 35 when changing from binary to ternary mixtures. 

The current results therefore confirm the permeation mechanism already observed in previous works, whereby permeability is strongly affected by the value of RH, and more importantly excludes any negative interaction among acidic gases in the polymers. Both H_2_S and CO_2_ permeability are maintained when changing from pure components to ternary mixtures. Therefore, no competitive sorption between the two acids seems to exist in the membrane. As a matter of fact, as reported by Berlinger et al. [36], the pH of hydrated PFSA film decreases to the same extent that the water content in the materials increases due to the presence of the -SO_3_H side group. Thus, it is likely that neither CO_2_ nor H_2_S are completely dissociated in the water channel due to the acidity of the environment [17,37,38]; therefore, they permeate through the membrane as solvated molecules, with no direct interaction with the polymer [17]. This is indeed a very different situation with respect to other materials, such as the EDA-modified Nafion, which showed strong competitive sorption, as the permeability was mediated by the molecule sorption on the amine groups [32,39].

**Figure 5:**
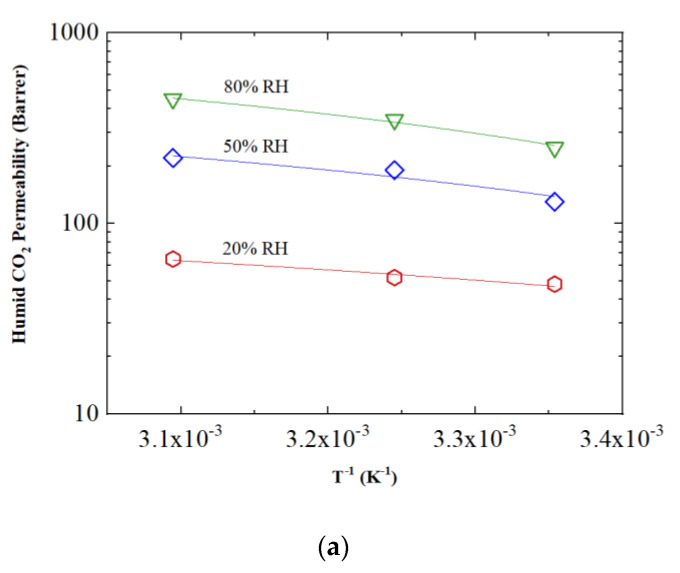

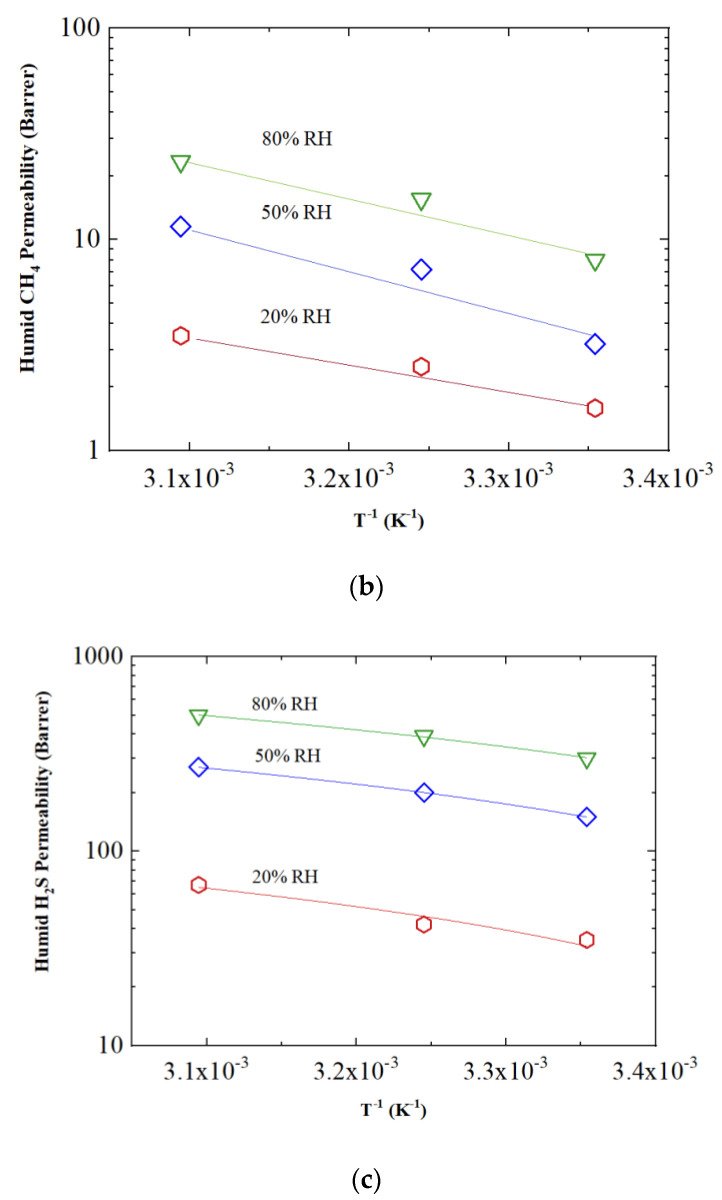
Humid permeability data as a function of the inverse of temperature for (**a**) CO_2_, (**b**) CH_4_ and (**c**) H_2_S.

The permeability values shown in Table 1 were then analyzed as a function of the inverse temperature to verify the actual dependence of the membrane on temperature and humidity. The results are reported in Figure 5, where an Arrhenius-type trend is followed by all penetrants at all of the different values of relative humidity. The determination coefficient R^2^ is always greater than 0.96, effectively demonstrating the linear relationship between the logarithm of permeability and the inverse of temperature. 

On the basis of the permeability data at the three different temperatures investigated, it is possible to calculate the activation energy for each gas of the mixture as a function of constant humidity using Equation (5):

(5) $$\;{\left(\frac{\partial \ln P_{i}}{\partial \left(\frac{1}{T}\right)}\right)}_{RH}=-\frac{E_{Pi}^{RH}}{R}$$

**Figure 6:**
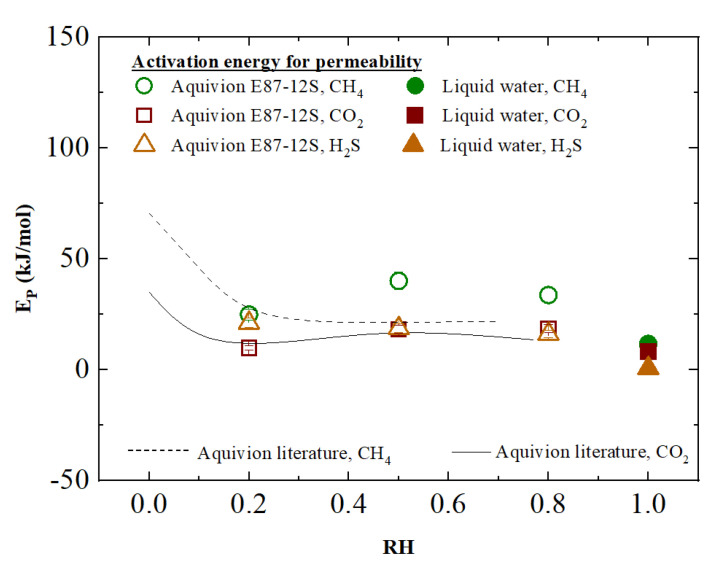
Experimental activation energy in Aquvion^®^ E87-12S as a function of relative humidity for CO_2_, H_2_S and CH_4_ compared to activation energy reported in the literature for CO_2_ (continuous black line) and CH_4_ (dashed black line) [19].

The experimental activation energies under humid conditions are plotted in Figure 6, providing a quantitative determination of the effect of temperature on the external polymer phase. The reported values are in line with those previously published for the same type of polymer [19,25], at least for CO_2_, while for CH_4_ they seem slightly higher, especially at the higher relative humidity inspected. Once again, there are no data available for pure H_2_S, but the values obtained are in line with those calculated for H_2_S/CH_4_ mixtures. In particular, the activation energy calculated for hydrogen sulfide exhibits values similar to those obtained for carbon dioxide, which has the same acidic behavior and a similar solubility in water.

Apart from that, it can be observed that activation energy remains largely constant at the values of relative humidity investigated in the present work, which is in line with what has been reported in literature, but is decreased with respect to the value obtained from the dry polymer. In addition, the described values are rather close to those prevailing in liquid water, confirming once again the mechanism controlling gas transport in hydrated Aquivion^®^.

### Effect of High Pressure on Mixed Gas Permeability

On the basis of the results obtained from the permeation tests described above, it was decided to proceed with two other tests with ternary mixtures at 35 °C and high pressure (10 bar), to verify the response of the membrane to acid gases at higher partial pressures. These operative conditions were chosen because they are the closest ones to those potentially found in real industrial natural gas sweetening systems that can be safely achieved in laboratory plants. Although the pressure was limited compared to the levels found in extraction plants, the high concentration of CO_2_ and especially of H_2_S in the mixture led to partial pressure values for these compounds that were close to that of the atmosphere. Tests were conducted following the same protocol used for low pressure tests; Equation (3) was used to estimate methane permeability, and Equations (1) and (2) were used to calculate the permeability of CO_2_ and H_2_S with the help of the gas composition in the downstream, which was controlled and verified using the GC. Only two values of relative humidity (50% and 90%) were considered for these tests, as these were the values of highest interest for hypothetical applications. 

**Figure 7:**
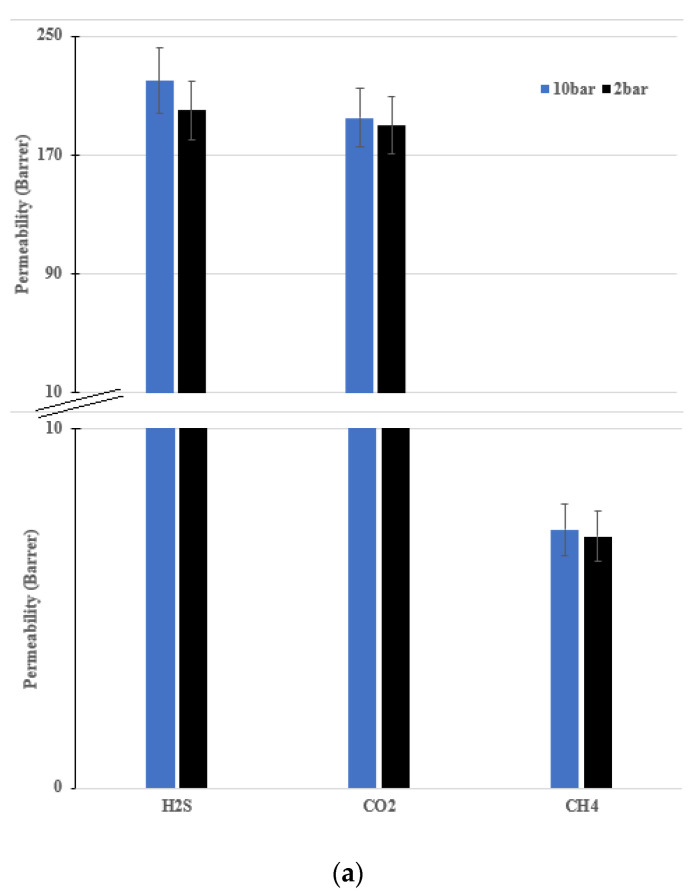

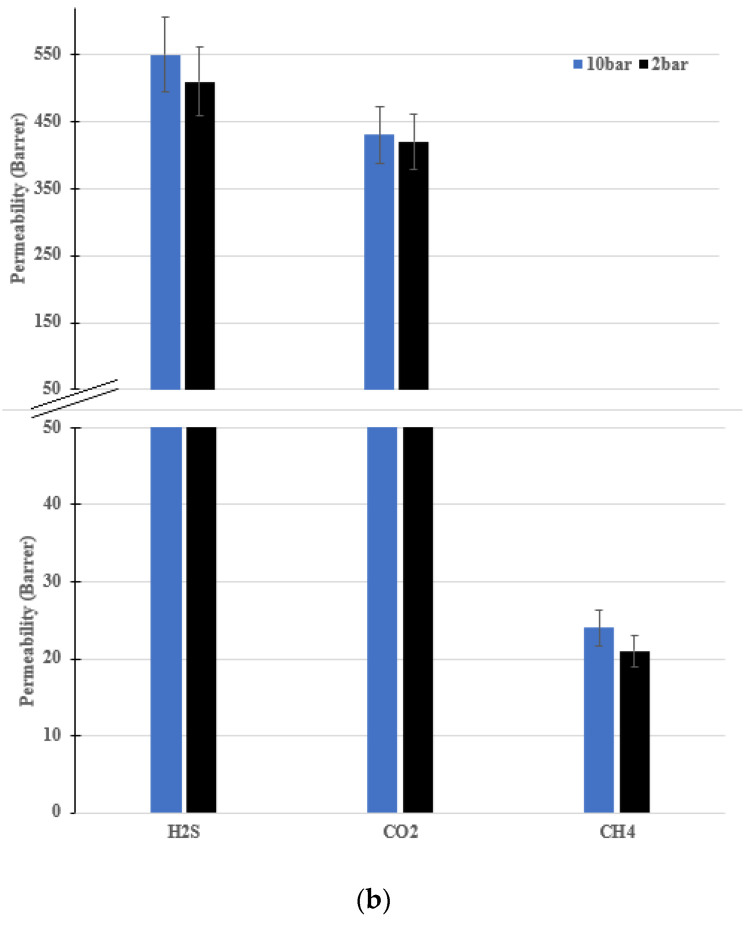
Comparison of permeability results between high- and low-pressure H_2_S, CO_2_ and CH_4_ at (**a**) RH50% and (**b**) RH90%.

The results are reported in Figure 7, in which the permeability obtained at high pressure is compared with data previously obtained at low (maximum 3 bar) upstream pressure. It can be clearly seen from the figures below that the pressure increase does not substantially change the permeability values of the three gases. This increment could be related to the plasticization effect caused by the high content of acid gases. However, the higher permeability obtained at a feed pressure of 10 bar is, in any case, very limited, while the ideal selectivity remains substantially unchanged, so that any plasticization problems can be considered negligible for this type of membrane application.

**Figure 8:**
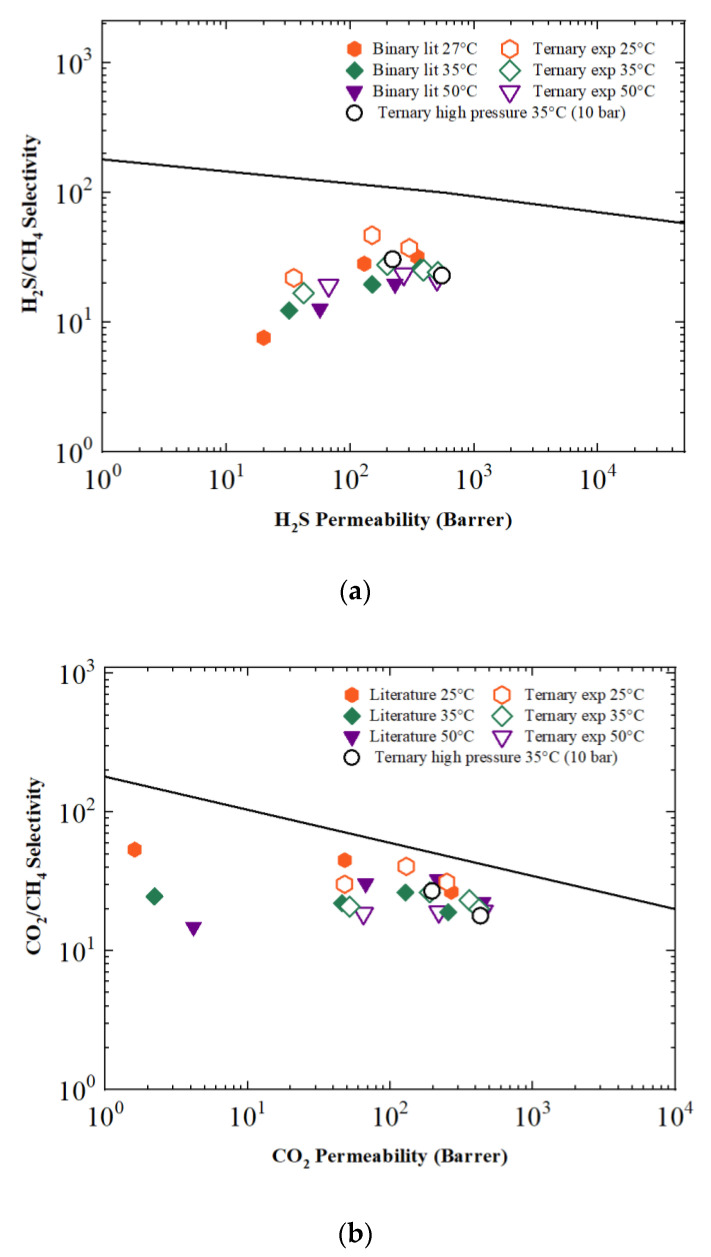
(**a**) H_2_S/CH_4_ Robeson plot [12] compared with the results obtained for the binary mixture [27]; (**b**) CO_2_/CH_4_ Robeson plot [15] compared with the literature data [25].

In fact, although the experimental data at 10 bar always present values higher than those of the previously described data, the differences are within the margin of error of the experimental data, which was on the order of 10%, as also reported in Figure 8 for the sake of completeness. 

It should be noted that, unlike what happens in other polymers or in the same perfluorosulfonate polymers in the absence of water [26], in this case, the increase in pressure does not correspond to a significant decrease in selectivity (as calculated from Equation (4)), which remains largely unchanged for the CO_2_/CH_4_ system and only drops slightly, from 25 to 23, for the H_2_S/CH_4_ system at RH90%, a variation that, in any case, can, as already mentioned, largely be attributd to experimental error. Therefore, even at high pressure, the Aquivion maintains its separation capabilities unchanged, and could potentially exhibit even better performance by virtue of the increased permeability of both H_2_S and CO_2_.

The potential of Aquivion^®^ for the purposes of gas separation is indeed mainly related to its ability to maintain its properties under a wide range of different operating conditions. Indeed, if compared with other polymers in the Robeson’s Plot, it is certainly not among the best-performing materials for the separation of either CO_2_/CH_4_ (Figure 8a) or—especially—H_2_S/CH_4_ (Figure 8b). Nonetheless, it is close to many other materials currently used in real-world applications related, for example, to natural gas sweetening [4], and certainly demonstrates very robust results even in very harsh conditions. It can be noted that selectivity is, in general, not much influenced by the changes in relative humidity and upstream pressure, confirming the high flexibility and chemical stability of this kind of PFSA in the field of gas separation. In this regard, it is also worth noting that, even if long-run tests could not be performed with the current experimental setup, some samples were tested continuously for more than 2 months, switching between pure and mixed gas tests, and no sign of degradation was observed.

## 4. Conclusions

In this paper, Aquivion^®^ E87-12S, a pefluorosulphonic acid polymer produced by Solvay Specialty Polymers, was tested with acid ternary mixture under different operating conditions. The feed stream composition was set to be equal to 10% CO_2_, 9% H_2_S and 81% CH_4_ in order to thoroughly assess this material for the purposes of acid gas separation and natural gas sweetening.

We aimed to investigate the membrane response when it was exposed to more than one acid gas under humid conditions, even at high feed pressures, in order to investigate the presence of any competitive sorption and to determine its potential for the simultaneous removal of both CO_2_ and H_2_S from CH_4_. Specifically, the Aquivion properties were investigated at three temperatures—25 °C, 35 °C and 50 °C—and at values of relative humidity ranging from 20% to 95%, since the separation performances of heterogenous PFSA increases with increasing temperature and water activity. Even the change in the upstream feed pressure was investigated in order to determine whether any plasticization conditions were occurring in the material.

On the basis of the results obtained, it is possible to state that the permeability of all three gases increased with increasing relative humidity, especially in the case of CO_2_ and H_2_S, which reached values equal to 440 Barrer and 520 Barrer, respectively, at 35 °C and RH95%. These values, as well as those observed for methane, were in the same range as those obtained during the pure gas permeation tests, thus suggesting that the simultaneous presence of the two acid gases in the feed does not affect the membrane permeation properties. 

The separation performances of Aquivion^®^ for CO_2_ and H_2_S are also positively affected by temperature and pressure. Indeed, the permeability increased with increasing temperature following an Arrhenius-type law, while selectivity was only slightly affected, due to the similarity among the different values of activation energy, especially when considering high humidity. Under these conditions, the values for all three compounds present in the mixture tended towards those found in liquid water.

Finally, even when increasing the feed total pressure to 10 bar, the properties of the membrane seemed to remain unchanged, with changes in permeability and selectivity remaining well inside the experimental uncertainty of the measurements. 

The semi-commercial Aquivion E87-12S material was therefore confirmed to be very attractive in the field of natural gas sweetening, as its performance was very stable, being only slightly affected by changes in the operating conditions.

## Figures and Tables

**Table 1:** Experimental permeability and selectivity of H_2_S, CO_2_ and CH_4_ in Aquivion^®^ at different temperatures and relative humidities.

T [°C]	P [Bar]	RH	Permeability H_2_S [Barrer]	Permeability CH_4_ [Barrer]	Permeability CO_2_ [Barrer]
25 °C	1.90	20%	35	1.59	48
25 °C	1.60	50%	150	3.2	130
25 °C	1.60	80%	300	8	250
35 °C	1.50	20%	42	2.5	52
35 °C	1.15	50%	200	7.2	190
35 °C	2.15	80%	390	15.5	350
35 °C	2.00	95%	520	21	440
50 °C	1.68	20%	67	3.5	65
50 °C	1.50	50%	270	11.5	220
50 °C	1.50	80%	500	23.4	450

## Data Availability

The raw data are available upon request by emailing the corresponding author.
